# Ökonomische Auswirkung der COVID-19-Pandemie in der Allgemein- und Viszeralchirurgie: Ein Vergleich von Leistungs- und Erlösdaten zweier Kliniken aus Krankenhäusern unterschiedlicher Versorgungsstufen

**DOI:** 10.1007/s00104-021-01448-z

**Published:** 2021-06-21

**Authors:** Johannes Binder, Maximilian Brunner, Matthias Maak, Axel Denz, Georg F. Weber, Robert Grützmann, Christian Krautz

**Affiliations:** 1 Klinik für Allgemein- und Viszeralchirurgie des, Universitätsklinikum Erlangen, Krankenhausstraße 12, Eingang Maximiliansplatz, 91054 Erlangen, Deutschland; 2 Chirurgische Abteilung, Kreiskrankenhaus St. Anna Höchstadt/Aisch, Spitalstraße 5, 91315 Höchstadt a. d. Aisch, Deutschland

**Keywords:** Krankenhausentlastungsgesetz, Coronavirus, Chirurgie, DRG-Abrechnungsdaten, Behandlungsqualität, Hospital Relief Act, Coronavirus, Surgery, DRG billing data, Treatment quality

## Abstract

**Hintergrund:**

Bereits während der ersten Welle der COVID-19-Pandemie wurden die deutschen Krankenhäuser dazu aufgefordert, ihre elektiven Operationskapazitäten einzuschränken, um eine Überlastung des Gesundheitswesens abzuwenden. Im März 2020 wurden mit dem COVID-19-Krankenhausentlastungsgesetz finanzielle Hilfen zum Ausgleich dieser Einschränkungen vereinbart. Die Auswirkungen dieser Maßnahmen wurden in dieser Studie regional untersucht.

**Material und Methoden:**

Es wurden die Leistungsdaten und Erlöskennzahlen der Klinik für Allgemein- und Viszeralchirurgie des Universitätsklinikums Erlangen (UKER) und der Chirurgischen Abteilung des Kreiskrankenhauses St. Anna Höchstadt/Aisch (KKH) im Zeitraum 01.04. bis 30.06.2019 mit denen des Jahres 2020 verglichen.

**Ergebnisse:**

Es zeigte sich eine deutliche Reduktion der Bettenauslastung und der Fallzahlen stationär behandelter Patienten. Letztere sanken um 20,06 % im UKER bzw. 60,76 % im KKH. Nichtonkologische elektive Operationen nahmen um 33,04 % (UKER) bzw. 60,87 % (KKH) ab. Die Anzahl der Notfalleingriffe blieb am UKER unverändert und verringerte sich am KKH um 51,58 %.Die Erlöse aus DRG („diagnosis-related groups“) sanken um 22,12 % (UKER) und 54 % (KKH). Nach Berücksichtigung der Ausgleichszahlungen und Einsparungen aus variablen Sachkosten verzeichnete das UKER einen Verlust von −3,87 %, das KKH erreichte hingegen einen positiven Erlöseffekt von 6,5 %.

**Diskussion:**

Die nichtselektive Einschränkung des elektiven Operationsbetriebs beeinflusste die Patientenversorgung und die Erlöskennzahlen an beiden Standorten signifikant. In Bezug auf die Schaffung von Intensivkapazitäten erscheint diese ungezielte Maßnahme jedoch nicht effizient. Zudem führte die einheitliche Freihaltepauschale zu einer unausgeglichenen Verteilung der finanziellen Hilfen zwischen den untersuchten Kliniken.

## Abstract (en)

**Background:**

During the first wave of the coronavirus disease 2019 (COVID-19) pandemic, German hospitals were required to limit the capacity for elective surgery to prevent the healthcare system from general overload. In March 2020, the German government passed the COVID-19 Hospital Relief Act that guaranteed compensation payments for these limitations. In this study the regional impact of this intervention were analyzed.

**Material and methods:**

The performance data and revenue figures for the departments of general and visceral surgery of the University Hospital of Erlangen (UKER) and the District Hospital St. Anna Höchstadt/Aisch (KKH) during the period from 1 April to 30 June 2019 were compared with the respective period in 2020.

**Results:**

There was a significant decrease in bed occupancy rates and case numbers of inpatient treatment. The latter declined by 20.06% in the UKER and 60.76% in the KKH. Nononcological elective surgery was reduced by 33.04% in the UKER and 60.87% in the KKH. The number of emergency procedures remained unchanged in the UKER, while they decreased by 51.58% in the KKH. The revenues from diagnosis-related groups (DRG) decreased by 22.12% (UKER) and 54% (KKH), respectively. After taking compensation payments and savings from variable material costs into account, the UKER recorded a loss of −3.87%, while there was a positive revenue effect of 6.5% in the KKH.

**Discussion:**

The nonselective restriction of elective surgery had a significant impact on patient care and revenue figures at both locations. With respect to the increase of intensive care capacities, such untargeted measures do not appear to be efficient. In addition, the fixed rate of compensation payments led to an unbalanced distribution of the financial aid between the two departments.

## Hintergrund und Fragestellung

Aufgrund hoher Infektionszahlen mit zum Teil schweren Krankheitsverläufen hat die COVID-19(„coronavirus disease 2019“)-Pandemie bereits früh zu erheblichen Schwierigkeiten in den Gesundheitssystemen einiger Länder geführt. Zur Vermeidung der drohenden Überlastung des Deutschen Gesundheitssystems wurden alle Krankenhäuser im März 2020 dazu aufgefordert, planbare Operationen und Eingriffe zu verschieben. Hierdurch konnten zusätzliche Kapazitäten und Ressourcen zur Behandlung von COVID-19-Patienten freigesetzt werden [3].

Speziell im Fokus der Kapazitätssteigerung und Ressourcenbündelung waren hierbei die Intensivstationen. In Bayern trat im Zuge dessen am 19.03.2020 eine Allgemeinverfügung auf Grundlage von § 28 Abs. 1 Satz 1 des Infektionsschutzgesetzes (IfSG) in Kraft. Demnach hatten unterschiedliche Einrichtungen der Patientenversorgung „bis auf Weiteres alle planbaren Behandlungen zurückzustellen oder zu unterbrechen“, um so zusätzliche materielle und personelle Ressourcen zu schaffen. Dennoch musste sichergestellt werden, dass es bei den weiterhin notwendigen Behandlungen zu keiner Einschränkung der Versorgungsqualität kam.

Neben den sich daraus ergebenden medizinischen Herausforderungen traten auch mögliche wirtschaftliche Risiken in den Fokus. Als Grundlage der Abrechnung stationärer Leistungen dient in Deutschland das G‑DRG(„diagnosis-related groups“)-System. Die Entscheidung zur Einschränkung des Normalbetriebes hatte somit zwangsläufig finanzielle Auswirkungen auf eine bereits stark im Wettbewerb stehende Krankenhauslandschaft zur Folge. Schätzungen, die während der Vorbereitung dieser Maßnahmen z. B. durch die Diakonie Deutschland und dem Deutschen Evangelischen Krankenhausverband (DEKV) durchgeführt wurden, gingen in einer Modellrechnung von Ausfällen in Millionenhöhe aus [10, 11]. Demnach ergäben sich für ein Krankenhaus mit 300 bis 400 Betten bei einem Wegfall von ca. 50 % der nicht dringlichen Behandlungen ein jährlicher Verlust von 12 Mio. €. Die Bundesregierung kündigte daher eine umfassende finanzielle Unterstützung an.

Am 27.03.2020 trat das Gesetz zum Ausgleich COVID-19-bedingter finanzieller Belastungen der Krankenhäuser und weiterer Gesundheitseinrichtungen („COVID-19-Krankenhausentlastungsgesetz“) in Kraft [6]. Das Gesetz sah umfangreiche Maßnahmen vor (s. Infobox 1). Unter anderem sollte eine bundeseinheitliche, tagesbezogene Freihaltepauschale in Höhe von 560 € für jedes freigehaltene Bett (in Bezug auf die durchschnittliche Belegung des Vorjahres) die Einnahmeausfälle der Krankenhäuser abfedern. Diese finanzielle Unterstützung war zunächst für den Zeitraum vom 16.03.2020 bis 30.09.2020 vorgesehen.

Die vorgelegten Maßnahmen unterlagen bereits zum Zeitpunkt ihrer Einführung einer breiten Diskussion. Dabei wurde vor allem die Zielgenauigkeit bzw. die Verteilungsungerechtigkeit der bundeseinheitlichen Freihaltepauschale kritisiert. Letztere wurde schließlich ab dem 13.07.2020 durch differenzierte Beträge in fünf Stufen zwischen 360 bis 760 € abgelöst. Nichtdestotrotz bleibt eine pauschalierte Zahlung vor dem Hintergrund höchst variabler Kostenstrukturen der Krankenhäuser allenfalls ein kurzfristiges Mittel. Auf der Krankenhausebene bleiben zudem die Unterschiede zwischen Fachrichtungen unberücksichtigt, was langfristig zu betriebswirtschaftlichen Spannungen innerhalb der Krankenhäuser führen kann.

Wie stark sich die Einschränkung des Regelbetriebes auf die Erlössituation in der Chirurgie auswirkt und welche Unterschiede in Bezug auf Krankenhausgröße, Behandlungsspektrum und Fallzahlen bestehen, ist weiterhin nur unzureichend beantwortet. Vor dem Hintergrund der zweiten Pandemiewelle und der weiterhin hohen Anzahl an Infektionen (2.134.936) und Todesfällen (51.870) in Deutschland (Stand: 24.02.2020) sind diese Fragen relevanter denn je [20].

### Infobox 1 Übersicht zu den finanziellen Hilfen aus dem COVID-19-Krankenhausentlastungsgesetz (Stand 27.03.2020)

- Freihaltepauschale in Höhe von 560 €
- Bonus für zusätzliche Intensivbetten in Höhe von 50.000 €
- Mehrkosten für Schutzausrüstung in Höhe von 50 € pro Patient
- Vorläufiger Pflegeentgeltwert erhöht auf 185 €
- Aussetzung des Fixkostendegressionsaufschlags
- Verringerung der Prüfquote auf 5 %
- Aussetzung der Abschläge bei Abrechnungskorrekturen

Ziel dieser Studie war es, die Auswirkungen der Einschränkung des Normalbetriebs und der finanziellen Hilfen des COVID-19-Krankenhausentlastungsgesetzes im Hinblick auf die Leistungsdaten und die Erlössituation in der Allgemein- und Viszeralchirurgie im direkten Vergleich zweier Kliniken aus unterschiedlichen Krankenhausversorgungsstufen zu analysieren.

## Studiendesign und Untersuchungsmethoden

Als Datengrundlage dienten bei dieser retrospektiven Studie die G‑DRG-Abrechnungsdaten aller stationären Fälle der Klinik für Allgemein- und Viszeralchirurgie des Universitätsklinikums Erlangen (UKER) sowie der Chirurgischen Abteilung des Kreiskrankenhauses Höchstadt (KKH). Das Universitätsklinikum Erlangen ist ein Krankenhaus der Maximalversorgung, während das Kreiskrankenhaus Höchstadt als Krankenhaus der Grundversorgung eingestuft ist. Die von der Politik vorgegebenen Einschränkungen wurden in den jeweiligen Krankenhäusern ab dem 19.03.2020 umgesetzt. Die fixe Pauschale für somatische Krankenhäuser wurde durch die COVID-19-Ausgleichszahlungs-Änderungs-Verordnung (AusglZÄV) vom 13.07.2020 durch fünf Ausgleichsstufen abgelöst [7]. Da die abrechnungsrelevanten Daten nicht tagesaktuell, sondern lediglich monatsweise zur Verfügung gestellt werden konnten, wurde für diese Studie als Beobachtungszeitraum der 01.04.2020 bis 30.06.2020 gewählt. Als Vergleichszeitraum dienen die Zahlen des entsprechenden Zeitraums im Jahr 2019. Die Daten eignen sich insofern zum direkten Vergleich, da die Versorgung chirurgischer Patienten des Kreiskrankenhauses Höchstadt, im Rahmen einer Kooperation, unter ärztlicher Leitung der Chirurgischen Klinik des Universitätsklinikums Erlangen erfolgt und daher gleiche Standards gelten.

Als Referenzzeitpunkt zum Einschluss wurde das Aufnahmedatum gewählt. Patienten, die über den Beobachtungszeitraum hinaus stationär behandelt wurden, wurden nicht ausgeschlossen. Die Bettenauslastung wurde auf Monatsbasis berechnet, wobei die tatsächliche mit der maximalen Bettenbelegung ins Verhältnis gesetzt wurde, welche sich aus dem Produkt der insgesamt verfügbaren Betten und der Anzahl der Kalendertage im Berichtszeitraum errechnet. Als Grundlage dienten die Planbetten der Normalstation, ohne zugehörige Intensivkapazitäten.

Die durchgeführten Operationen wurden in Operationskategorien (z. B. Adipositaschirurgie, hepatobiliäre Chirurgie etc.) zusammengefasst (s. Anhang Tab. 1). So wurden beispielsweise proktologische Operationen in der Kategorie „Proktologie“ zusammengefasst, wohingegen einzelne häufig durchgeführte Operationen (z. B. Cholezystektomie) eine eigene Kategorie bekamen. Zudem erfolgte eine Stratifizierung nach Alter, Geschlecht, Dringlichkeit (elektiv/Notfall), Aufnahmeart (Wiederaufnahme ja/nein), Indikationsart (onkologisch/nichtonkologisch), Zugangsweg (offen/laparoskopisch/roboterassistiert/retroperitoneoskopisch), Verweilweildauer und Art der Entlassung (regulär/externe Verlegung/gestorben). Im Weiteren wurden der Case Mix (CM), der Case-Mix-Index (CMI), die Erlöse und die UGDV(untere Grenzverweildauer)-Abschläge berechnet. Letztere sind eine Kennzahl, die sich durch Absenkung der bundeseinheitlichen Prüfquote des Medizinischen Dienstes der Krankenversicherung (MDK) gemäß dem COVID-19-Krankenhausentlastungsgesetz erlösrelevant ändern könnte. Obwohl die Einhaltung der Verweildauern den MDK per se nicht zur Abrechnungsprüfung berechtigt, werden insbesondere Krankenhausbehandlungen an der unteren Grenzverweildauer häufig geprüft [14, 24].

Um eine Vergleichbarkeit der Erlöse zu gewährleisten, wurden die Erlöse im Jahr 2020 ohne Preissteigerung der Entgelte berechnet. Daher wurde für 2019 und 2020 der gleiche Landesbasisfallwert (3560,54 €) verwendet. Zudem wurden die für das Jahr 2019 zugrunde liegenden Daten unter Verwendung des sog. Übergangsgrouper 2019 bis 2020 um die Pflegepersonalkosten bereinigt, da diese ab 2020 nicht mehr im DRG-System abgebildet werden.

Zur Berechnung der Höhe der Freihaltepauschalen wurde die Anzahl der im Jahresdurchschnitt 2019 voll- oder teilstationär behandelten Patienten auf einen Zeitraum von drei Monaten kondensiert und hiervon die Zahl der Belegungstage im Untersuchungszeitraum 2020 abgezogen. Eine positive Differenz wurde mit der tagesbezogenen Pauschale von 560 € multipliziert. Die Zuschläge in Höhe von 50 € für zusätzliche Schutzausrüstung pro Fall wurden ebenfalls mit der entsprechenden Fallzahl multipliziert. Die Erhöhung des Pflegeentgeltwerts auf 185 € wurde ebenfalls nicht in die Analyse eingeschlossen, da die Ausgliederung der Pflegekosten erst im Jahr 2020 wirksam wurde und für das Jahr 2019 keine Definition der Pflegepersonalkosten bzw. keine Vereinbarung der Pflegebudgets vorlagen. Die Aufhebung des Fixkostendegressionsabschlages für das Jahr 2020 wurde nicht berücksichtigt.

Zum Zeitpunkt der Erhebung lagen die Kostendaten beider Krankenhäuser nicht abschließend vor, somit musste auf eine detaillierte Kosten- und Erlösgegenüberstellung verzichtet werden. Für die Fixkosten wurden daher für das Jahr 2020 angenommen, dass sich diese zu 2019 nicht verändert haben. Für die variablen Sachkosten wurde pauschal ein Betrag in Höhe von 15 % der Entgelte je Fall zugrunde gelegt. Dieses Vorgehen wurde bereits von Augurzky et al. angewandt [1]. Basierend auf diesen vereinfachten Annahmen wurde die Erlössituation anschließend kalkuliert. Hierbei setzen sich die Bruttoerlöse aus den Erlösen ohne Pflegekosten zuzüglich der Summe aus der Freiheitspauschale und den Zusatzentgelten für Schutzmaßnahmen zusammen. Die Nettoerlöse ergeben sich aus den Bruttoerlösen zuzüglich der Differenz aus den variablen Sachkosten. Bei Einsparungen aufgrund nicht angefallener variabler Sachkosten liegt somit ein positiver Erlöseffekt vor.

Die o. g. Daten wurden bei normalverteilten, kontinuierlichen Daten mittels ungepaartem T‑Test analysiert. Binäre oder kategoriale Daten wurden mittels χ^2^-Test oder dem Exakten Test nach Fisher analysiert. Die Daten wurden als absolute und relative Häufigkeiten bzw. als Mittelwerte mit Standardabweichungen dargestellt. Das Signifikanzniveau (α) wurde auf 5 % festgelegt (zweiseitig).

## Ergebnisse

### Auswirkungen auf die Bettenauslastung, Fallzahlen und Behandlungsqualität

Die Chirurgische Klinik des Universitätsklinikums Erlangen hatte in den Jahren 2019 und 2020 insgesamt 63 Planbetten zur Verfügung. Für das Kreiskrankenhaus Höchstadt (KKH) wurden 5 Planbetten für die Allgemein- und Viszeralchirurgie als Berechnungsgrundlage verwendet. Hierbei muss beachtet werden, dass die tatsächliche Anzahl an Planbetten in Höchstadt tatsächlich höher wäre, aufgrund von Umbauarbeiten diese jedoch nicht voll genutzt werden konnten. Zudem liegt der Schwerpunkt der chirurgischen Versorgung im Bereich der Unfallchirurgie und Orthopädie. Im Vergleich zu den Vorjahresmonaten ist die Bettenauslastung in Erlangen von 93 % auf 74 %, in Höchstadt von 79 % auf 36 % gesunken. Die Fallzahlen stationär behandelter Patienten sanken im Betrachtungszeitraum von 718 im Jahr 2019 auf 574 im Jahr 2020 (UKER) bzw. von 79 auf 31 (KKH).

Insgesamt wurden im Betrachtungszeitraum 2019 am UKER 623 und am KKH 77 Operationen durchgeführt. Demgegenüber war 2020 in beiden Krankenhäusern ein deutlicher Rückgang der Operationszahlen zu verzeichnen. Am UKER kam es zu einem Rückgang um 24,24 % auf 472 Operationen, am KKH sogar um 54,55 % auf 35. Die Entwicklung der Fallzahlen entsprechend den Operationskategorien ist in Tab. 1 im Anhang dargelegt. Entgegen dem Gesamttrend fanden sich in einzelnen Kategorien Ausreißer (z. B. „multiviszerale Resektionen“ +100 %), welche jedoch auf niedrige Fallzahlen zurückzuführen sind.

Die Stratifizierung der Eingriffe nach Dringlichkeit ergab in Erlangen eine signifikante Abnahme der elektiven Operationen (−33,04 %) bei fast unveränderter Anzahl an Notfalleingriffen (−1,71 %), was zu einer signifikanten Zunahme des Notfallanteils im Jahr 2020 führte (*p* < 0,004; Tab. 1). Gleichfalls sank die Anzahl der onkologischen Eingriffe im Vergleich zu den nichtonkologischen Operationen deutlich weniger (−3,77 %).

Surrogatparameter für die Behandlungsqualität wie z. B. Verweildauer, Wiederaufnahmerate und Mortalitätsrate unterlagen am UKER keinen signifikanten Veränderungen. Am Grundversorger (KKH) kam es zu einem deutlichen Rückgang von Elektiv- und Notfalleingriffen (−60,9 % vs. −51,5 %), sodass sich der relative Anteil zwischen den Dringlichkeitsstufen nicht änderte (*p* = 0,442; Tab. 2). Onkologische Operationen werden u. a. aufgrund der Nähe zum Maximalversorger (Darmkrebs- und Pankreaskarzinomzentrum, Sarkomzentrum, Leberzentrum und Hautkrebszentrum Erlangen) in Höchstadt nicht durchgeführt, womit der Vergleich zu nichtonkologischen Eingriffen entfiel. Interessanterweise wurden im Beobachtungszeitraum 2020 signifikant mehr offene und weniger laparoskopische Eingriffe im Vergleich zum Vorjahreszeitraum durchgeführt (*p* = 0,033). Analog zum Maximalversorger wurden die Surrogatparameter für die Behandlungsqualität (Verweildauer, Wiederaufnahmerate und Mortalitätsrate) in Höchstadt ebenfalls nicht durch die Einschränkungen des Normalbetriebes beeinflusst.

**Table 1:** 

		Jahr				*p*-Wert^a^
		2019		2020		
Alter (Jahre)		55,1	±18,4	55,2	±17,0	0,93
Geschlecht	Männlich	350	56,2 %	268	56,8 %	0,843
	Weiblich	273	43,8 %	204	43,2 %	
Dringlichkeit	Elektiv	448	71,9 %	300	63,6 %	0,004
	Notfall	175	28,1 %	172	36,4 %	
Art der Indikation	Nichtonkologisch	464	74,5 %	319	67,6 %	0,009
	Onkologisch	159	25,5 %	153	32,4 %	
Zugangsweg	Offen	449	72,1 %	362	76,7 %	0,336
	Laparoskopisch	160	25,7 %	101	21,4 %	
	Roboterassistiert	11	1,8 %	8	1,7 %	
	Retroperitoneoskopisch	3	0,5 %	1	0,2 %	
Verweildauer (Tage)		8,4	±11,1	8,67	±10,1	0,75
Wiederaufnahme	Nein	598	96,0 %	459	97,2 %	0,26
	Ja	25	4,0 %	13	2,8 %	
Art der Entlassung	Regulär	598	96,0 %	457	96,8 %	0,744
	Externe Verlegung	14	2,2 %	9	1,9 %	
	Verstorben	11	1,8 %	6	1,3 %	
*Gesamt*		*623*	*100,0* *%*	*472*	*100,0* *%*	*–*

^a^χ^2^-Test bzw. Fisher-Exakt-Test bei Feldfallzahl < 5. Alter und Verweildauer als Mittelwert (± Standardabweichung)

**Table 2:** 

		Jahr				*p*-Wert^a^
		2019		2020		
Alter (Jahre)		54,1	±17,4	58,9	±18,6	0,186
Geschlecht	Männlich	51	66,2 %	21	60,0 %	0,523
	Weiblich	26	33,8 %	14	40,0 %	
Dringlichkeit	Elektiv	54	70,1 %	27	77,1 %	0,442
	Notfall	23	29,9 %	8	22,9 %	
Art der Indikation	Nichtonkologisch	77	100,0 %	35	100,0 %	–
	Onkologisch	–	–	–	–	
Zugangsweg	Offen	53	68,8 %	31	88,6 %	0,033
	Laparoskopisch	24	31,2 %	4	11,4 %	
	Roboterassistiert	0	0,0 %	0	0,0 %	
	Retroperitoneoskopisch	0	0,0 %	0	0,0 %	
Verweildauer (Tage)		5,8	±8,1	10,0	±12,4	0,073
Wiederaufnahme	Nein	73	94,8 %	30	85,7 %	0,135
	Ja	4	5,2 %	5	14,3 %	–
Art der Entlassung	Regulär	76	98,7 %	32	91,4 %	0,09
	Externe Verlegung	1	1,3 %	3	8,6 %	
	Verstorben	0	0,0 %	0	0,0 %	
*Gesamt*		*77*	*100,00* *%*	*35*	*100,00* *%*	*–*

^a^χ^2^-Test bzw. Fisher-Exakt-Test bei Feldfallzahl < 5. Alter und Verweildauer als Mittelwert (± Standardabweichung)

### Auswirkungen auf die Erlöskennzahlen

**Figure 1:**
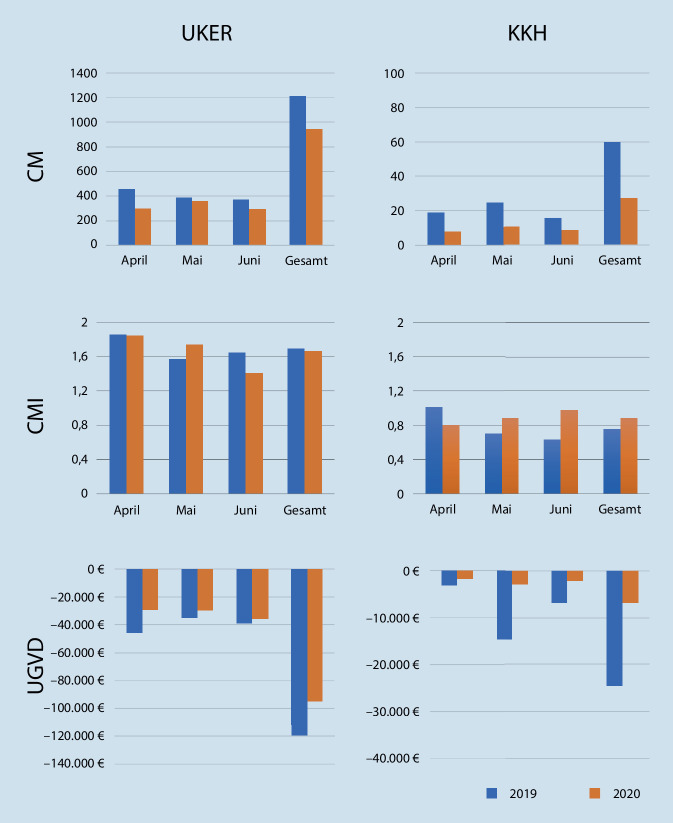

Die Einschränkung des Normalbetriebes hatte in beiden Kliniken teils erhebliche Auswirkungen auf die Erlöskennzahlen (Abb. 1). Aufgrund des geringeren Patientenaufkommens nahmen der Case Mix und die Erlöse statistisch signifikant ab. Dieser Einbruch war in Erlangen mit −22,1 % geringer als am KKH (−54 %; Tab. 3). Der CMI des UKER zeigte für den Monat Mai einen leichten Anstieg, wohingegen der CMI im Juni wieder sank. Ein ähnlicher Anstieg wurde zu Beginn der Einschränkungen bereits an einem anderen universitären Maximalversorger beobachtet [8]. Im Mittel kam es jedoch zu keiner statistisch signifikanten Änderung der Fallschwere in Erlangen (CMI: 1,66 vs. 1,69; *p* = 0,43). Der CMI am KKH nahm von 0,76 im Jahr 2019 auf 0,89 im Jahr 2020 zu. Diese Veränderung war jedoch nicht statistisch signifikant (*p* = 0,59).

Die Höhe der UGVD-Abschläge nahm am UKER zwar signifikant ab (*p* = 0,01), in Relation zum DRG-Erlös ergab sich jedoch keine wesentliche Änderung (2019: 2,77 % vs. 2020: 2,76 %). Mögliche Einspareffekte waren hier mit 211,95 € vernachlässigbar. Demgegenüber änderte sich die absolute Höhe der UGVD-Abschläge für das KKH nicht signifikant. Es fand sich aber in Relation zum DRG-Erlös ein deutlicher Rückgang (2019: 11,56 % vs. 2020: 6,97 %), mögliche Einsparungen durch gesunkene UGVD-Abschläge belaufen sich auf 4484,41 €.

**Table 3:** 

		Erlangen	Höchststadt
Erlöse aus DRG	2019	4.323.083 €	212.450 €
	2020	3.366.615 €	97.737 €
*Differenz zu 2019*	*–*	*−22,1* *%*	*−54,0* *%*
Bruttoerlöse^a^	2019	4.323.083 €	212.450 €
Bruttoerlöse	2020	4.012.506 €	209.047 €
Erlöse aus DRG		3.366.615 €	97.737 €
Freihaltepauschale		609.991 €	109.564 €
Zulagen		35.900 €	1.550 €
*Differenz zu 2019*	*–*	*−7,2* *%*	*−1,6* *%*
Nettoerlöse	2019	4.323.083 €	212.450 €
Nettoerlöse	2020	4.155.977 €	226.254 €
Bruttoerlöse		4.012.507 €	209.047 €
Einsparungen aus variablen Sachkosten		143.470 €	17.207 €
*Differenz zu 2019*	*–*	*−3,9* *%*	*6,5* *%*

*DRG* „diagnosis-related groups“

^a^Entspricht den Erlösen aus DRG 2019

### Veränderung der Erlössituation durch finanzielle Hilfen

Unter Einbeziehung der Freihaltepauschalen sowie der Zulagen für Schutzausrüstung änderte sich die Erlössituation deutlich zugunsten des Grundversorgers (Tab. 3). Während die Bruttoerlöse des UKER um 7,2 % sanken, zeigte sich in Höchstadt nur noch ein Rückgang von 1,6 %. Nach Berücksichtigung der Einsparungen aus variablen Sachkosten (Nettoerlöse) ergab sich sogar ein positiver Erlöseffekt von 6,5 % für das KKH, während die Nettoerlöse des UKER noch 3,9 % unter den Vorjahreszahlen lagen. Um den Effekt der abgestuften Freihaltepauschalen zu simulieren, wurden die Bruttoerlöse mit der jeweiligen abgestuften Freihaltepauschale erneut berechnet. Hierbei fand sich für das UKER ein positiver Erlöseffekt von 1,17 % und ein Erlösrückgang zum Vorjahr von −2,8 % für das KKH.

## Diskussion

Die von der Politik beschlossenen Einschränkungen wurden an beiden betrachteten Krankenhäusern umgesetzt. Die Auswirkungen auf die Fallzahlen sowie auf die Erlössituation waren im Vergleich sehr unterschiedlich. So sind am KKH die Fallzahlen überproportional stark gesunken. Besonders hervorzuheben ist hier die Abnahme der Notfalleingriffe um 51,6 %. In diesem Zusammenhang könnte zu befürchten sein, dass es hier eventuell zu Einschränkungen in der Notfallbehandlung gekommen ist. Slagmann et al. zeigten, dass es während der COVID-19-Pandemie im Frühjahr 2020 in Deutschland einen signifikanten Rückgang medizinischer Notfälle gab [21]. Entsprechend dieser Entwicklung fand sich in einer aktuellen populationsbasierten Analyse in Deutschland eine signifikant geringere Anzahl von Patienten mit unkomplizierter Blinddarmentzündung. Diese Studie zeigte jedoch auch, dass sich die Häufigkeit, die Behandlungsrate und die Komplikationsrate der komplizierten Blinddarmentzündung nicht unterschied [18]. Inwieweit eine Reduktion notfallmäßiger Krankenhausaufnahmen und Eingriffe tatsächlich zu einer Einschränkung der Gesundheitsversorgung bzw. zu Gesundheitsgefährdung in Deutschland geführt hat, bleibt an dieser Stelle unklar.

Im Laufe der Pandemie wurde deutlich, dass Patienten auch aufgrund von Ängsten vor einer Ansteckung mit SARS-CoV‑2 („severe acute respiratory syndrome coronavirus type 2“) medizinische Einrichtungen meiden. Eine Folge der COVID-19-Pandemie könnte somit sein, dass onkologische Erkrankungen nicht oder erst in einem späteren Stadium entdeckt werden [13, 17]. Zudem könnte es aufgrund von Vermeidungsstrategien dazu kommen, dass weniger aggressive (effektivere) Therapieoptionen bevorzugt empfohlen werden [15]. Solche Entwicklungen wären jedoch hochproblematisch, da diese mit einer signifikanten Reduktion an gewonnenen Lebensjahren einhergehen [23]. In den von uns betrachteten Kliniken konnten wir keine Hinweise auf eine Therapieverzögerung finden. Laut einer nationalen Umfrage gaben jedoch 34 % der Befragten an, dass sie aufgrund der Einschränkung des Normalbetriebs auch Verzögerungen in der onkologischen kolorektalen Chirurgie erwarten [2]. Diese Befürchtungen wurden später in einer bundesweiten Erhebung der Eingriffszahlen durch den Expertenbeirat des Bundesgesundheitsministeriums untermauert. Letzterer wies einen Rückgang von 12 % im ersten Halbjahr 2020 nach [1].

Die Einschränkung des Krankenhausregelbetriebs und der Operationskapazitäten sollte initial vor allem der Schaffung von Intensivkapazitäten zur Behandlung von COVID-19-Patienten dienen. Da diese Patienten im Rahmen des COVID-19-assoziierten Lungenversagens u. a. hochkomplexe intensivmedizinische Krankheitsbilder entwickeln, ist eine suffiziente Behandlung oft nur in spezialisierten Einrichtungen mit einer geeigneten Infrastruktur möglich. Vor diesem Hintergrund soll an dieser Stelle die Effizienz der bundesweiten, nichtselektiven Reduktion von Operationskapazitäten im stationären Sektor anhand der vorliegenden Daten hinterfragt werden. So sanken im betrachteten Zeitraum die Fallzahlen im KKH im Vergleich prozentual deutlich stärker. Somit konnte hier im Gegensatz zum Universitätsklinikum relativ gesehen eine höhere Entlastung intensivmedizinischer Behandlungskapazitäten erreicht werden. Da trotz moderner Ausstattung in Höchstadt dennoch die intensivmedizinischen Möglichkeiten nicht vergleichbar mit denen eines Universitätsklinikums sind, mussten im Verlauf COVID-19-Intensivpatienten verlegt werden (z. B. fehlende Möglichkeit einer extrakorporalen Membranoxygenierung [ECMO] etc.). Demgegenüber blieb die Anzahl der onkologischen Operationen am UKER mit exzellenter intensivmedizinischer Infrastruktur unverändert (bundesweit konnte sogar eine leichte Zunahme an hochkomplexen Krebsoperationen registriert werden [1]). Dies führte wahrscheinlich dazu, dass wiederum Kapazitäten auf den Intensivstationen gebunden waren und damit dem initialen Ziel der Schaffung von Intensivkapazitäten entgegenstanden. In den unterschiedlichen Phasen der Pandemie gab es auch unterschiedliche Auslastungen der Intensivstationen. Im Falle einer Überbelastung müssen alle freiwerdenden Kapazitäten auch in kleineren Krankenhäusern genutzt werden. Während Phasen der Entspannung bei sinkender Auslastung sollte in den Augen der Autoren die intensivmedizinische Behandlung schwerkranker COVID-19-Patienten zur Sicherung einer optimalen Versorgung, wenn möglich, in Krankenhäusern der Maximalversorgung erfolgen. Somit könnte die ungezielte Einschränkung der Operationskapazitäten hier aus den genannten Gründe fehlgeleitet sein.

Auch wenn die flächendeckende, ungezielte Einschränkung des Krankenhausregelbetriebs und der Operationskapazitäten nicht effizient war, so war sie dennoch effektiv. Ab der 12. Kalenderwoche sank die durchschnittliche Anzahl von Patienten auf Intensivstationen in Deutschland [1]. Zudem zeigen die vorliegenden Daten, dass die Einschränkungen auch ohne eine negative Beeinflussung der Behandlungsqualität (gemessen an Surrogatparametern wie z. B. Verweildauer, Wiederaufnahmerate und Mortalitätsrate) vollzogen werden können.

Zu Beginn der Pandemie führten Stöß et al. eine nationale Umfrage unter den Ordinarien der deutschen Universitätskliniken der Allgemein- und Viszeralchirurgie durch [22]. Die geschätzten Veränderungen der Case-Mix-Punkte und der Erlöse wurden durchschnittlich mit −26 % ± 12 % bzw. −28 ± 12 % angegeben, was jeweils etwas höher ist als der in Erlangen beobachtete Rückgang. Die befragten Ordinarien gingen zudem von einer deutlichen Abnahme der Fallschwere (CMI = −20 ± 17 %) aus. Eine logische Erklärung dieser Erwartung ergibt sich möglicherweise aus der Tatsache, dass Intensivbetten für chirurgische Patienten nur noch eingeschränkt zugänglich waren und somit komplexe Operationen mit einem höheren Risiko für eine mögliche Intensivpflicht nicht in unveränderter Frequenz durchgeführt werden konnten. In Erlangen blieb der CMI unverändert. Diese Stabilität der Fallschwere lässt sich unter anderem auf einen relativ hohen Anteil an onkologischen und notfallmäßigen Eingriffen zurückführen (zusammen fast 50 % aller Eingriffe). In diesem Kontext ist die Entwicklung der bundesweiten Zahlen viszeralchirurgischer Operationen interessant. Wie oben bereits erwähnt, lässt sich hier eine gewisse Priorisierung hochkomplexer Eingriffe erkennen [1]. Eine Priorisierung hochkomplexer Eingriffe könnte für Kliniken höherer Versorgungsstufen aber auch eine Möglichkeit zur Stabilisierung von CMI und Erlösen sein. Auf diese Weise erfüllen die Kliniken dann zwar die gesundheitspolitischen Vorgaben zur Reduktion von Operationskapazitäten, erreichen aber mit hoher Wahrscheinlichkeit nicht eine maximale Entlastung der Intensivstationen. Im Vergleich zu der erwarteten und beobachteten Entwicklung der Erlöskennzahlen im universitären Maximalversorger reduzierten sich der CM und die DRG-Erlöse im Grundversorger Höchstadt um 54 %. Der CMI des KKH blieb statistisch unverändert. An dieser Stelle zeigt sich, dass kleine Krankenhäuser – wie das KKH – ohne die finanziellen Hilfen des COVID-19-Krankenhausentlastungsgesetzes besonders stark von der Einschränkung des Krankenhausregelbetriebs getroffen werden.

Die Zusammensetzung der Brutto- und Nettoerlöse (Tab. 3) zeigt, dass die chirurgische Klinik des Krankenhauses der unteren Versorgungsstufe zu Beginn der Pandemie möglicherweise überproportional vergütet wurde. Legt man die abgestuften Freihaltepauschalen unseren Berechnungen zugrunde, so zeigt sich für beide Versorgungsstufen eine wesentlich ausgeglichenere Verteilung der Hilfen. Die Anpassungen der fixen Freihaltepauschale sind somit als wichtige Entscheidung zu werten. Der Expertenrat sah jedoch noch im August 2020 eine „Verlängerung der finanziellen Hilfen nach § 21 KHG […] über den 30. September 2020 hinaus […]“ als nicht erforderlich an. Vielmehr solle ein individueller Ausgleich mit den Kostenträgern vereinbart werden [4]. Aufgrund der aktuellen zweiten Pandemiewelle wurden die abgestuften Freihaltepauschalen für den Zeitraum vom 18.11.2020 bis einschließlich 31.01.2021 wieder eingeführt. Finanzielle Hilfen für Krankenhäuser sind demnach weiter vorgesehen. Die Auszahlung erfolgt jedoch gezielter anhand der 7‑Tages-Inzidenz (> 70) sowie der Zahl der freien Intensivbetten (< 25 %) an den Krankenhäusern, die mindestens die Notfallstufe 2 abdecken müssen [5, 16]. Da viele Krankenhäuser aktuell unter einem starken wirtschaftlichen Druck stehen, wird dieses Vorgehen bereits kritisiert [9, 12, 19]. Im Hinblick auf die fehlende Effizienz der flächendeckenden Einschränkung des Regelbetriebs erscheint eine Selektion der Auszahlung und somit eine gezielte, bedarfsgerechte Einschränkung jedoch wünschenswert.

## Limitationen

Die Ergebnisse der Studie sind unter Berücksichtigung einiger Limitationen zu betrachten. Aufgrund der fachspezifischen Auswahl mit Auswertung von lediglich zwei Kliniken sind die Ergebnisse nicht repräsentativ für das Fachgebiet und auch nicht übertragbar auf das deutsche Gesundheitssystem als Ganzes. Hierfür sollte eine groß angelegte retrospektive Analyse der Abrechnungsdaten erfolgen. Da keine Daten zu ambulanten Einweisungen vorliegen, kann auch keine Aussage über eine Dynamik in diesem Bereich getroffen werden. Die Beurteilung der Sachkosten fand vereinfacht nach der etablierten Methode des Expertenrats statt. Daher können mögliche Änderungen der Kostenstruktur auch hier nicht detailliert abgebildet werden (z. B. durch Reduktion teurer Technologien o. Ä.). Eine Aussage über die intensivmedizinische Behandlung von COVID-19-Patienten sollte und kann aus dieser Studie ebenfalls nicht getroffen werden.

## Schlussfolgerung

Während der ersten Pandemiewelle hat die bundesweite Einschränkung des Regelbetriebs zu einer effektiven Schaffung von Intensivkapazitäten geführt. Die Effizienz dieser ungezielten Maßnahme sollte jedoch, im Hinblick auf eine optimale Versorgungsqualität, hinterfragt werden. Darüber hinaus könnte die fixe Freihaltepauschale mit einer ungerechten Verteilung zwischen chirurgischen Kliniken unterschiedlicher Versorgungsstufen verbunden sein. Die Einführung der abgestuften Freihaltepauschalen führt möglicherweise auch in der Allgemein- und Viszeralchirurgie zu einer höheren Verteilungsgerechtigkeit. Trotz dieser Anpassung können ohne eine individuelle Berücksichtigung der lokalen Gegebenheiten jedoch Finanzierungsengpässe entstehen.

## Anhang

**Table 4:**

Indikationsart	Operationskategorien	Jahr (UKER)		Differenz	Gesamt	Jahr (KKH)		Differenz	Gesamt
		2019	2020			2019	2020		
Elektiv	Adipositaschirurgie	11	7	−36,36 %	18	–	–	–	0
	Appendektomie	2	0	−100,00 %	2	–	–	–	0
	Cholezystektomie	17	13	−23,53 %	30	4	2	−50,00 %	6
	Dünndarmresektion	4	6	50,00 %	10	–	–	–	0
	Endokrine Chirurgie	17	3	−82,35 %	20	–	–	–	0
	Hepatobiliäre Chirurgie	17	23	35,29 %	40	–	–	–	0
	Hernienchirurgie	27	12	−55,56 %	39	24	14	−41,67 %	38
	Kolorektale Chirurgie	87	52	−40,23 %	139	1	0	−100,00 %	1
	Lymphadenektomie	3	11	266,67 %	14	–	–	–	0
	Melanom Chirurgie	29	21	−27,59 %	50	–	–	–	0
	Milz	3	3	0,00 %	6	–	–	–	0
	Multiviszerale Resektion	2	4	100,00 %	6	–	–	–	0
	Ösophagus‑/Magenchirurgie	30	14	−53,33 %	44	–	–	–	0
	Pankreaschirurgie	27	15	−44,44 %	42	–	–	–	0
	Proktologie	105	57	−45,71 %	162	12	4	−66,67 %	16
	Sarkomchirurgie	19	19	0,00 %	38	–	–	–	0
	Sonstiges	11	15	36,36 %	26	10	4	−60,00 %	14
	Stomaanlage/Resektion	37	25	−32,43 %	62	3	2	−33,33 %	3
	*Gesamt*	*448*	*300*	*−33,04* *%*	*748*	*54*	*26*	*−51,85* *%*	*81*
Notfallmäßig	Appendektomie	37	35	−5,41 %	72	3	2	−33,33 %	4
	Cholezystektomie	19	20	5,26 %	39	1	1	0,00 %	2
	Dünndarmresektion	11	17	54,55 %	28	1	0	−100,00 %	1
	Hepatobiliäre Chirurgie	1	0	−100,00 %	1	–	–	–	0
	Hernienchirurgie	9	6	−33,33 %	15	4	0	−100,00 %	4
	Kolorektale Chirurgie	21	17	−19,05 %	38	1	0	−100,00 %	1
	Milz	0	3	300,00 %	3	–	–	–	–
	Ösophagus‑/Magenchirurgie	4	3	−25,00 %	7	–	–	–	–
	Pankreaschirurgie	1	1	0,00 %	2	–	–	–	–
	Proktologie	39	35	−10,26 %	74	–	–	–	–
	Sarkomchirurgie	2	2	0,00 %	4	–	–	–	–
	Sonstiges	19	24	26,32 %	43	13	6	−53,85 %	19
	Stomaanlage/Resektion	12	9	−25,00 %	21	–	–	–	0
	*Gesamt*	*175*	*172*	*−1,71* *%*	*347*	*23*	*8*	*−60,87* *%*	*31*

*UKER *Universitätsklinikums Erlangen, *KKH *Kreiskrankenhauses St. Anna Höchstadt
